# Gender-specific interpersonal neural synchronization during same-gender closeness-building interactions among strangers: an fNIRS hyperscanning study

**DOI:** 10.1186/s40359-026-05129-6

**Published:** 2026-08-10

**Authors:** Yuhang Li, Shibo Zhou, Liutong Ou, Yilin Hu, Xiuyun Lin, Qi Zhao, Rihui Li, Peilian Chi

**Affiliations:** 1 Department of Psychology, Faculty of Social Sciences, University of Macau, Macau SAR, 999078 China; 2 Centre for Cognitive and Brain Sciences, Institute of Artiﬁcial Intelligence and Brian Sciences, University of Macau, Macau SAR, 999078 China; 3 Department of Biomedical Engineering, College of Engineering, Wichita State University, Kansas, Kansas United States; 4 Chengdu Shuangqing Primary School, Chengdu, 610051 China; 5 Institute of Developmental Psychology, Faculty of Psychology, Beijing Normal University, Beijing, 100875 China; 6 School of Psychology, Hainan Normal University, Haikou, 571158 China; 7 Department of Biomedical Engineering, Faculty of Engineering, University of Macau, Macau, SAR Macau China

**Keywords:** Hyperscanning, Gender, Friendship, Relationship-building, FNIRS

## Abstract

**Background:**

Self-disclosure, sharing of personal information, is crucial for initiating and deepening interpersonal relationships. Although it has been consistently shown that mutual self-disclosure fosters closeness between strangers, the underlying dual brain neural mechanism remains unexplored. This study aims to uncover these gender-modulated differences and their underlying neural mechanisms.

**Method:**

We investigated the interpersonal neural synchronization (INS) among zero-history, same-gender strangers. We applied the functional near-infrared spectroscopy (fNIRS) hyperscanning technique to simultaneously measure the brain hemodynamic activities of 32 female and 30 male dyads. Participants engaged in an adapted Fast Friends paradigm, featuring a naturalistic interaction progression from superficial "small talk" to incrementally escalating levels of mutual self-disclosure.

**Results:**

The structured interactions elicited significant increases in self-disclosure, emotional arousal, and perceived interpersonal closeness as the tasks progressed from the no-disclosure stage to increasingly intimate self-disclosure stages. This psychological escalation was accompanied by significantly enhanced INS between the dorsolateral prefrontal cortex (DLPFC) and temporoparietal junction (TPJ) exclusively during the deep self-disclosure stage relative to the no-disclosure stage. Crucially, gender significantly moderated the relationship between this cross-regional coupling and relationship-building outcomes (closeness: β = -0.386, p_FDR = .025). For female-female dyads, INS significantly mediated the relationship between self-disclosure and closeness (indirect effect = 0.03); however, these associative and mediation pathways were entirely absent in male-male dyads.

**Conclusion:**

These findings illuminate the dual-brain neural underpinnings of relationship initiation, highlighting that relationship formation between females may rely more heavily on emotional verbal expression and INS than their male peers. This advances theoretical models of social cognition and offers insights into developing strategies for various social settings.

**Supplementary Information:**

The online version contains supplementary material available at https://doi.org/10.1186/s40359-026-05129-6.

## Background

Close relationships are fundamental to human experience, with self-disclosure playing a critical role in fostering relational closeness [1, 2]. Despite its importance, the neural mechanisms underlying escalating mutual self-disclosure in naturalistic interactions remain poorly understood. Prior neuroimaging studies primarily relied on single-brain recordings within established relationships, neglecting the interpersonal neural coupling during relationship initiation [3, 4]. To address this gap, we employed hyperscanning to elucidate the dyadic neural processes of closeness-building in same-gender strangers. Given divergent neurobiological foundations and social expectations across genders, relationship-building dynamics and self-disclosure depth are posited to differ significantly between male-male and female-female dyads [5, 6]. Consequently, this study aims to uncover gender-modulated differences in interactive behaviors and their underlying neural mechanisms during relationship development.

### Self-disclosure and closeness building

Self-disclosure is an empirically established mechanism for enhancing interpersonal closeness [1, 7]. To examine relationship formation among strangers, researchers developed th*e Fast Friends Procedure:* a structured interaction paradigm featuring incrementally escalating self-disclosure levels [8, 9]. This progression ranges from superficial exchanges ("small talk") to intimate revelations about personal values, attitudes, and beliefs [10].Through such disclosure, individuals share their inner selves, fostering mutual understanding and relational bonds [11, 12].

Neuroimaging studies further elucidate self-disclosure’s role in social interaction, revealing activation in the mesolimbic dopamine system during disclosure, suggesting an intrinsic reward value that reinforces interpersonal closeness [3, 4]. However, these findings derive from artificial laboratory settings devoid of naturalistic social interaction. Moreover, single-brain recording approaches cannot capture the dynamic interaction between the brains in real-life social conversation. Consequently, brain imaging during ecologically valid social interactions remains necessary to fully explicate self-disclosure’s role in relationship formation and its neural underpinnings.

Hyperscanning allows for simultaneous dual-brain recording, revealing that interpersonal neural synchronization (INS) increases with social engagement and relationship depth, such as in parent–child or friend dyads [13–17]. However, most studies examined existing relationships, leaving the neural synchronization dynamics during relationship-building among strangers unclear. By examining zero-history dyads, we aim to uncover how INS develops, advancing the understanding of brain-to-brain communication mechanisms in trust and empathy formation.

### Gender-specific dynamics

Besides the depth of self-disclosure, gender may also influence the interaction dynamics during closeness development. Compared to cross-gender relationships, same-gender relationships exert an earlier and more enduring influence on lifelong development [18–20]. In adolescence and early adulthood, same-gender friendship significantly relates to psychological well-being [21], resilience [22]and the construction of personality [23]. While the interaction between opposite-gender dyads (e.g., dating) gained much attention from researchers [24, 25], the influential factors of friendship between the same gender were unclear. Thus, we focused on same-gender friendship in the current study.

Prior studies have suggested substantial differences between male-male and female-female relationships [6, 26, 27]. Women typically engage more in intimate disclosures, while men’s relationship relies more on shared activities [4, 27–29]. Evolutionary perspectives suggest that females historically relied on communal tending and emotional sharing to facilitate reciprocal support [30, 31], whereas males focused on competitive, agentic cooperation where vulnerability might signal a lack of dominance [32, 33]. Supported by neurobiological findings on oxytocin-mediated behaviors [29, 34], these biological foundations and contemporary social norms suggest that gender may significantly shape self-disclosure patterns and relationship formation mechanisms [5, 35–37].

Indeed, hyperscanning studies have shown gender-specific neural activation patterns during cooperative tasks, with distinct regions being more active in female versus male dyads. Baker et al. (2016) found female-female dyads exhibited task-related INS in the right temporal cortex, while significant INS in male-male dyads occurred in the right inferior prefrontal cortex. Zhang et al. (2017) found that female dyads, but not male dyads, showed increased inter-brain coherence in the left TPJ. However, the impact of gender on INS during closeness-building among same-gender individuals has not been thoroughly explored, presenting an opportunity to delve into the neurobiological mechanisms that underpin these gender-specific communication processes.

### The current study

The present study aimed to investigate the dyadic neural mechanisms of self-disclosure in building closeness among same-gender dyads, with a lens of gender. We engaged same-gender participants in a Fast Friend procedure with step-wisely increasing self-disclosure depth. At the same time, we recorded the hemodynamic activities of their brains simultaneously by the fNIRS-based hyperscanning technique. We aimed to investigate how self-disclosure modulates INS and whether these effects were distinctly manifested across male and female dyads. We hypothesized that higher INS and interpersonal closeness ratings should be observed as the depth of self-disclosure increases. Also, INS during this process was hypothesized to serve as a statistical mediator linking self-disclosure and interpersonal closeness. As for the gender difference, we hypothesized that the male-male group should exhibit lower interpersonal closeness increase and a weaker relationship between self-disclosure and interpersonal closeness. This research advances theoretical understanding of neural foundations in relationship initiation while elucidating gender's role in social cognition, ultimately enriching models of interpersonal relationship initiation among unacquainted same-gender individuals.

## Method

### Participants

We recruited 32 pairs of female-female and 30 male-male zero-history strangers through advertising in the university. The ages for the female and male groups were 23.06 ± 3.11 and 22.44 ± 2.81 years. Participants are at least 18 years old, have normal or corrected-to-normal vision, and are right-handed to ensure consistency in brain lateralization. The sample size was determined based on previous hyperscanning studies, which typically employ 20–30 dyads per group to detect reliable effects [38–41]. Written informed consent was obtained from all the participants. The study protocol was approved by the Research Ethics Panel of the University of Macau (Ref: SSHRE20-APP006-FSS).

### Tasks and procedures

**Fig. 1:**
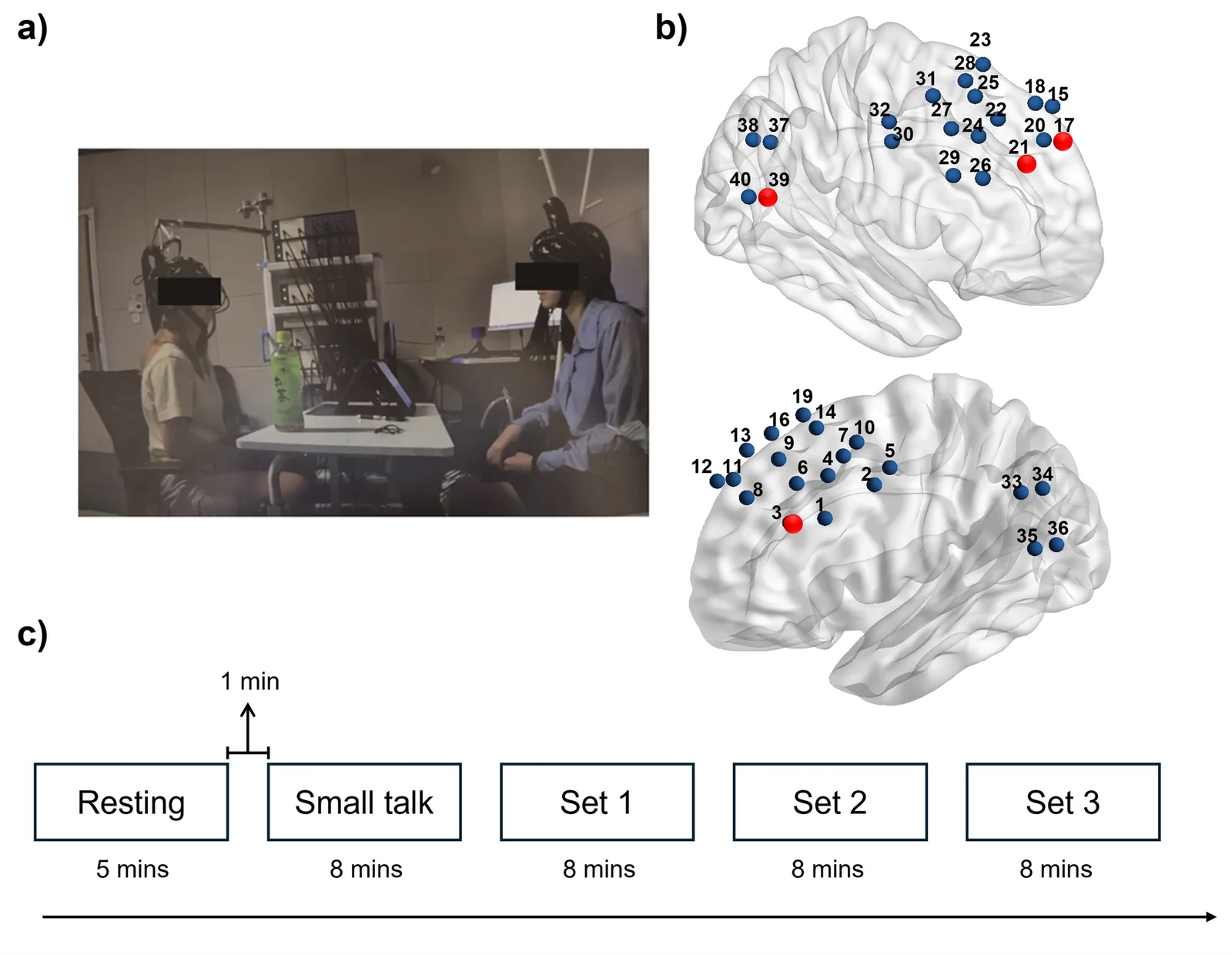
The Flow Chart of Study Procedure. **a** The experimental environment and setting. **b** Spatial configuration of the 40 fNIRS measurement channels on the cortical surface, with all channel numbers explicitly labeled to correspond with Table 1. The red-colored circles highlight the key channels of interest (CH3, CH17, CH39, and CH21) that demonstrated significant task-evoked INS increases in subsequent statistical analyses. **c** The experimental procedure demonstration

Participants were randomly paired and participated in the Fast Friends paradigm, adapted to enhance closeness through escalating self-disclosure [9, 42]. The participants were required to sit face-to-face on both sides of a table (Fig. 1A). After obtaining consent, participants completed initial psychological questionnaires while the fNIRS apparatus was being set up. Each dyad commenced the experiment with a 5-min resting-state scan, during which they were instructed to keep their eyes closed and remain motionless. Subsequently, they engaged in naturalistic interactions based on provided topics, divided into four sets: one small talk task and three sets of closeness-building self-disclosure tasks, with escalating self-disclosure depth [9] (Fig. 1C). Each set of interactions lasted for eight minutes with one-minute intervals. During these one-minute intervals, participants were required to quietly complete the state psychological questionnaires on their screens regarding the interaction stage they had just finished. No informal or unscripted communication was allowed during this time, ensuring a controlled transition between tasks. Both participants in the dyads were required to talk about their answers regarding the given topics.

The Small Talk task focused on topics commonly discussed in casual settings, such as hobbies or general interests, and deliberately avoided eliciting intimate or personal information. An example topic from the Small Talk task is “*What's the best gift you've ever received?”* The self-disclosure tasks started with relatively neutral topics and progressively moved toward more personal and revealing questions. The sample topics in the closeness-building task are Set 1: “*Who do you want to invite if you can have dinner with anyone in the world?”*; Set 2: “*What’s the biggest accomplishment in your life so far?”*; Set 3: “*Tell the person what you like about him/her and need to say things you might not say to someone you've just met*.”

### Psychological measures

We collected a set of state questionnaires to assess participants' responses to the interaction and their interaction partners. The questionnaires measured the psychological state of participants regarding four aspects: the depth of self-disclosure, interpersonal closeness, emotional arousal, and the desire for further interaction. During the experiment, questionnaires were answered four times: after Small Talk, after Set 1, after Set 2, and after Set 3. The interpersonal closeness and emotional arousal were additionally measured before Small Talk.

#### Self-disclosure

To assess the depth of self-disclosure within each dyad, we used four items to measure the depth of participants’ own self-disclosure and four items to measure the depth of partners’ self-disclosure. Specifically, the four items evaluating the participant's own self-disclosure depth were: (1) I revealed a large amount of personal information during the conversation, (2) The information I disclosed was highly intimate and deep, (3) I was completely honest and authentic in what I shared, and (4) I expressed my true and deep core feelings. Correspondingly, the four items evaluating the perceived partner's self-disclosure depth were symmetrically phrased, replacing “I” with “my partner”. All items were rated on a 7-point Likert scale (1 = strongly disagree, 7 = strongly agree) [43].

Then the scores were averaged into a composite score for self-disclosure, representing the depth of self-disclosure for each dyad. The Cronbach’s alpha of the own and other self-disclosure is .71 and .83.

#### Interpersonal closeness

Interpersonal closeness was measured by two questions. The first question was the Inclusion of Other in the Self (IOS) Venn diagram [44]. Participants were required to choose the seven pairs of circles that best described their relationships with their interactive partners. The answers were coded as points 1 to 7. The second item is a self-reported question on closeness: *How close do you feel toward your partner?* The participants answered this question with points from 1 to 7. The two items were averaged for a composite score with a Pearson correlation coefficient of .68. The interpersonal closeness scores for both participants were averaged as the score for the dyads.

#### Emotional arousal

Self-Assessment Manikin Scale (SAMS) [45] was used to measure the level of emotional arousal in participants' interaction scenarios. The original version of SAMS consisted of three questions from three dimensions: valence, arousal, and dominance. Only one question from the arousal dimension was utilized in the experiment. Participants were instructed to use a slider to indicate their current level of arousal based on five pictures. The pictures range from a fully awake figure with a large star-like figure across its chest to a sleeping figure. The emotional arousal scores for both participants were averaged as the score for the dyads.

#### The desire for further interaction

Two items measured the participants’ desire for future interaction with their paired partners. One item asked the participant to rate how much they would like to spend time with their paired partner in the future. The other item asked participants to rate the likelihood of spending time with the paired partner in the future. The Pearson correlation coefficient of the two items is .74. The desire for further interaction for both participants was averaged as the score for the dyads.

### fNIRS data acquisition

An optical topography system (OxyMon LS, Artinis Medical Systems BV, Einsteinweg, Netherlands) was used to collect fNIRS data. Three sets of customized optode probes were placed bilaterally to cover frontal, temporoparietal junction (TPJ) cortices, and we placed 32 channels in the PFC and 8 channels in the TPJ for a total of 40 channels for each participant (Fig. 1B). The absorption of fNIRS light at three wavelengths (780, 805, and 830 nm) was measured at a sampling rate of 50 Hz. To confirm the anatomical position of each optode, magnetic resonance imaging was obtained using a 3.0T Siemens MAGNETOM Prisma MRI scanner from two typical participants (male and female). Based on fMRI localization, we identified brain regions corresponding to the channels and the hemispheres in which they are located (Table 1).

**Table 1:** Channel corresponds to brain areas

Channel	Brain Area	Hemisphere
1	Pars Triangularis Broca's Area	L
2–4	Dorsolateral Prefrontal Cortex	L
5	Pre-Motor and Supplementary Motor Cortex	L
6–9	Dorsolateral Prefrontal Cortex	L
10	Includes Frontal Eye Fields	L
11–13	Dorsolateral Prefrontal Cortex	L
14	Includes Frontal Eye Fields	L
15	Dorsolateral Prefrontal Cortex	R
16	Includes Frontal Eye Fields	L
17–18	Dorsolateral Prefrontal Cortex	R
19	Includes Frontal Eye Fields	L
20–22	Dorsolateral Prefrontal Cortex	R
23	Includes Frontal Eye Fields	R
24	Dorsolateral Prefrontal Cortex	R
25	Includes Frontal Eye Fields	R
26	Pars Triangularis Broca's Area	R
27	Dorsolateral Prefrontal Cortex	R
28	Includes Frontal Eye Fields	R
29	Pars Triangularis Broca's area	R
30	Pre-Motor and Supplementary Motor Cortex	R
31	Dorsolateral Prefrontal Cortex	R
32	Pre-Motor and Supplementary Motor Cortex	R
33	Supramarginal Gyrus Part of Wernicke's Area	L
34	Angular Gyrus	L
35–36	Temporoparietal Junction	L
37	Supramarginal Gyrus Part of Wernicke's Area	R
38	Angular Gyrus	R
39–40	Temporoparietal Junction	R

### fNIRS data analysis

#### Preprocessing

Data analysis utilized the NIRS Analyzer toolbox within MATLAB in conjunction with a customized MATLAB-based script [46]. During preprocessing, the first and last 30 s of data in each task were removed to obtain data during a steady state. The data were then downsampled to 10 Hz to reduce computation time. Then, Temporal Derivative Distribution Repair (TDDR) was conducted to detect and correct motion artifacts [47]. Using the modified Beer-Lambert law, we obtained the changes in oxyhemoglobin (HbO) and deoxyhemoglobin concentrations (HbR) for each channel. To eliminate global physiological systemic noise, a spatial Principal Component Analysis (PCA) filter was applied with a variance removal threshold set at 80% [48–51]. Finally, following previous studies [52], we excluded data above 0.8 Hz to avoid the aliasing of high-frequency physiological noise such as cardiac activity (∼0.8 to 2.5 Hz). We also excluded data below 0.01 Hz to remove very low-frequency fluctuations. Previous studies have shown that HbO is the most sensitive indicator of changes in regional cerebral blood flow and has the highest signal-to-noise ratio in fNIRS measurements [53]. Therefore, we only focused on changes in HbO concentration. The preprocessed HbO signals for each dyad were segmented into five stages: resting state, Small Talk, Self-Disclosure Set-1, Self-Disclosure Set-2, and Self-Disclosure Set-3.

#### Interpersonal neural synchronization(INS)

The INS was calculated using the wavelet transform coherence (WTC) [54], utilizing the *coherence* function in MATLAB 2023a. The INS ranges from 0 to 1, with a higher score indicating a higher level of INS. For each pair of time series, we generated a WTC matrix containing two dimensions: time and frequency. The WTC matrices were averaged across time for each task (resting state and four talk tasks). We calculated the WTC of 1600 (40*40) paired channels during resting and four task states, excluding channels with bad signals. Since no specific roles were designated to participants during the interactions, WTC values for reciprocal channel pairs (e.g., CH1-CH2 and CH2-CH1) were averaged, leading to 820 unique channel pairs. Computationally, for any given asymmetrical channel pair (e.g., CH3 in left DLPFC and CH21 in right DLPFC), the final INS value was defined as the average coherence of Participant A's CH3 with Participant B's CH21, and Participant B's CH3 with Participant A's CH21, ensuring a non-directional, dyadic representation of reciprocal multi-regional coupling [50]. This approach produced a two-dimensional matrix for every channel pair and documented INS values over all time points. WTC calculations were performed for every possible pairing of channels, and the resulting INS values were then averaged over the time points within each epoch.

#### Selection of frequency and channel of interest

We identified the frequency bins and channel pairs that demonstrated increased INS during conversational activities in comparison to resting states. For each channel and each frequency bin, pairwise t-tests were performed to compare INS values between resting states and each of the four talk tasks. To minimize false positives during this open-ended discovery phase, the significance threshold was strictly guarded using the Bonferroni correction method applied across the entire multidimensional channel-by-frequency space. Crucially, a channel pair and its corresponding continuous frequency bins were finalized as 'of interest' if the INS significantly enhanced during at least one of the four active conversational tasks relative to the resting state, ensuring that the feature selection was orthogonal to and independent of downstream behavioral correlations. Through this mask-generation procedure, the frequency band of interest was localized to a narrow continuous spectrum of 0.205–0.217 Hz, and exactly 46 unique functional cross-brain channel pairs survived the threshold. For these 46 locked pathways, INS values were averaged across the localized frequency band in the following analysis. A comprehensive index of these 46 baseline-responsive pathways is detailed in Supplementary Table S2.

### Statistical analyses

Statistical analyses of self-reported measurements and INS were performed using SPSS 25.0 with an alpha value of 0.05 (IBM, New York, NY, USA). We conducted repeated measures ANOVAs to assess the effect of the task stages and gender group on the self-reported mental states, including the depth of self-disclosure, the emotional arousal, the interpersonal closeness, and the desire for future interaction. The task condition was treated as a within-subject variable, and the gender group was treated as a between-subject variable. Post-hoc multiple comparisons with Bonferroni correction and simple effects analysis were performed as needed.

Next, we evaluated whether the structured task stages significantly enhanced INS compared to Small Talk. We performed pairwise t-tests between the INS of the Small Talk stage and each of the three closeness-building talk stages, applying the False Discovery Rate (FDR) correction with the Benjamini–Hochberg method to correct for significance across all channel pairs of interest.

Following this, for channel pairs that demonstrated a significant increase in INS due to the structured talk stages, we computed the increase in INS relative to the resting stage as ΔINS. Then we examined the relationship between behavioral measures and ΔINS. We employed Linear Mixed Models (LMMs) using the ‘fitlm’ function in MATLAB Statistics and Machine Learning Toolbox to analyze the behavioral data. The behavioral measurements, namely, self-disclosure, interpersonal closeness, emotional arousal, and desire for further interaction, were input as dependent variables in the model, and the ΔINS and gender groups as predictors. The fixed effects of gender and ΔINS were assessed with the dyads' ID input as a random factor. To investigate the moderation effect of gender on this relationship between ΔINS and behavioral measurements, the interaction between gender groups and ΔINS, and the random effect of dyads were also included in the model. Finally, we examined the mediation model with self-disclosure as the independent variable, interpersonal closeness as the dependent variable, and the ΔINS value as the mediator. The indirect effect was assessed using the bootstrap method with a repetition of 1000 times. The 95% confidence interval was also given accordingly. The moderate effect of gender group was also assessed in the mediation model. Specifically, we set the gender as a fixed factor for all three pathways in the mediation model and assessed the gender effects on these pathways.

## Results

### Escalated closeness by mutual self-disclosure

**Fig. 2:**
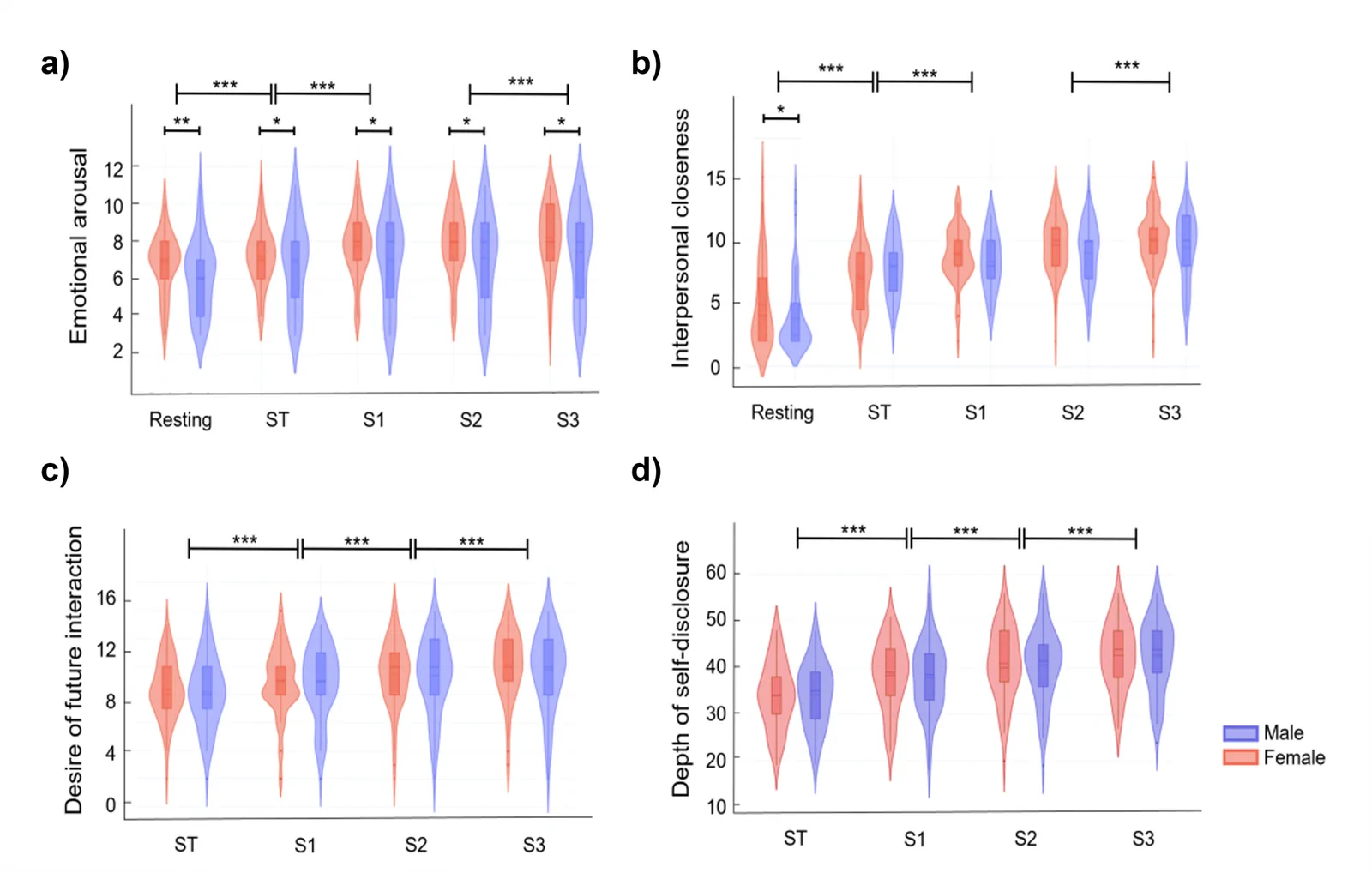
Effects of Gender and Self-Disclosure Stages on Behavioral Measures. This figure shows the results of repeated measures ANOVA for (**a**) Emotional arousal, **b** Interpersonal closeness, **c** Desire for further interaction, and **d** Depth of self-disclosure. The significant results of the paired t-test between the adjacent task stages and the effect of gender within each task stage are marked in the figure. Note: *: *p* <.05; **: *p* <.01; ***: *p* <.001. ST = Small Talk, s1 = Set1, s2 = Set2, s3 = Set3

A total of 124 valid questionnaires were collected (*N* = 124, male = 60, female = 64). We found significant effects of task conditions on all four dimensions of behavioral measurements (Depth of self-disclosure: *F*_(3, 120)_ = 127.81, *p* < 0.001, η^2^ = .60; Interpersonal closeness: *F*_(4, 119)_ = 125.88, *p* < .001, η^2^ = .75; Emotional arousal: *F*_(4, 119)_ = 28.69, *p* < .001, η^2^ = .39; Desire of further interaction: *F*_(3, 120)_ = 58.73, *p* < .001, η^2^ = .47) (Fig. 2 and Table 2). Post-hoc analysis revealed that among any two successive talk tasks, the ones that followed exhibited a significant rise in all four behavioral measurements (*p* < .001 for all), except for the comparison between Set 1 and Set 2 in terms of emotional arousal (*p* = .177) (Fig. 2A) and interpersonal closeness (*p* = .243) (Fig. 2B).

**Table 2:** The main effects of task conditions and the interaction effect of Task* Gender

Item	Gender	Set (Mean ± SD)					Task		Task* Gender	
		Resting	ST	S1	S2	S3	*F*	*p*	*F*	*p*
Disclosure	Male		34.11 ± 6.73	37.90 ± 7.42	40.61 ± 7.78	42.70 ± 7.72	127.81	<.001***	0.85	.467
	Female		34.07 ± 7.22	38.41 ± 7.24	41.04 ± 7.62	42.82 ± 7.04				
Further Interaction	Male		8.23 ± 2.52	8.97 ± 2.54	9.43 ± 2.60	9.88 ± 2.72	58.73	<.001***	0.54	.658
	Female		8.40 ± 2.14	9.08 ± 2.45	9.54 ± 2.43	10.25 ± 2.40				
Closeness	Male	3.81 ± 2.74	7.94 ± 2.38	8.34 ± 2.26	9.02 ± 2.36	9.55 ± 2.56	125.88	<.001***	16.58	<.001***
	Female	5.05 ± 3.23	7.28 ± 2.47	8.83 ± 2.28	9.66 ± 2.24	10.24 ± 2.23				
Arousal	Male	4.16 ± 1.93	4.80 ± 2.34	5.00 ± 2.33	5.19 ± 2.46	5.51 ± 2.47	28.69	<.001***	2.47	.062
	Female	4.86 ± 1.61	5.26 ± 1.57	5.79 ± 1.62	5.97 ± 1.79	6.21 ± 1.88				

Disclosure = Depth of self-disclosure, Further Interaction = Desire for Further Interaction, Closeness = Interpersonal closeness; N = 124 (Male = 60, Female = 64). ST = Small talk, S1 = Set1, S2 = Set2, S3 = Set3

We found significantly higher overall self-reported emotional arousal levels in the female group than in the male group (*F*_(1, 119)_ = 5.62, *p* = .019, η^2^ = .04). The simple effect analysis showed that the effect of gender group was significant before Small Talk (*p* = .009), after Set 1 (*p* = .031), after Set 2 (*p* = .018), and after Set 3 (*p* = .028) (Fig. 2A). The significant interaction effect between gender groups and task conditions was only found in interpersonal closeness (F_(4, 119)_ = 16.58, *p* < .001, η2 = .20) (Fig. 2B and Table 2). The simple effect analysis found that the female group has significantly higher interpersonal closeness than the male group only after Small talk (*p* = .023) but not after other sections of talk.

### Increased inter-brain synchronization within the DLPFC-TPJ synchronization induced by closeness-building conversation

**Fig. 3:**
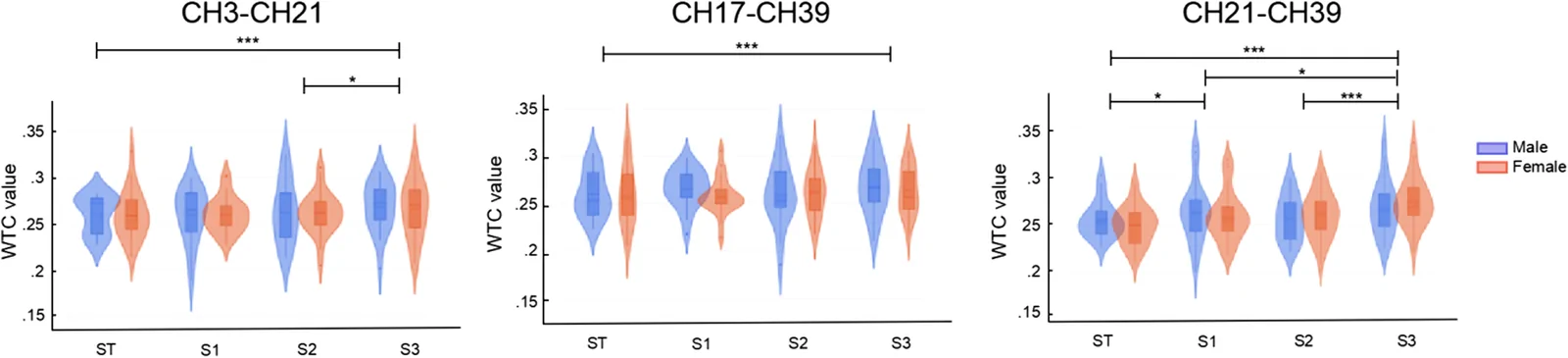
The INS changes along with the task conditions. The pair-wise t-test results identified significant differences in INS for specific channel pairs: CH3-CH21, CH17-CH39, and CH21-CH39. The significant gender effects within each task condition were also tested and marked in the figure (CH17-CH39). Note: *: *p* <.05; **: *p* <.01; ***: *p* <.001. ST = Small Talk, s1 = Set1, s2 = Set2, s3 = Set3

By comparing the WTC of resting states and task states, we selected 0.205 Hz-0.217 Hz as the frequency of interest and 46 channel pairs of interest. Based on the frequency of interest and channel pairs of interest, we detected the significant INS change along with the talk by comparing the INS of Set1-3 to Small Talk. After FDR correction, we only observed a significant rise in the INS for the Set 3 task compared to the Small Talk task across three channel pairs: CH3-CH21(*t*_(61)_ = 2.89, *p_*_*FDR*_ = .035), CH17-CH39 (*t*_(61)_ = 2.70, *p_*_*FDR*_ = .039), and CH21-CH39 (*t*_(61)_ = 4.02, *p_*_*FDR*_ = .002). CH3, 17, and 21 are all in DLPFC, and Ch39 corresponds to the TPJ. The details of corresponding brain regions can be found in Fig. 3.

We further assessed the INS change between each pair of tasks for these three channel pairs without correction. The results are shown in Fig. 3 and Table 3

**Table 3:** the pair-wise comparison of INS between tasks in selected channel pairs

Taks contrasts	CH3-CH21		CH17-CH39		CH21-CH39	
	t-value	*p*-value	t-value	*p*-value	t-value	*p*-value
ST vs Set 1	−1.008	.159	−1.357	.090	−2.177	.017*
ST vs Set 2	−0.709	.241	−0.987	.164	−1.106	.137
ST vs Set 3	−2.885	.003**	−2.704	.004**	−4.025	<.001***
Set 1 vs Set 2	0.327	.628	0.068	.527	1.037	.848
Set 1 vs Set 3	−1.518	.067	−1.320	.096	−1.838	.035*
Set 2 vs Set 3	−1.962	.027*	−1.125	.133	−2.716	.004*

*N* = 124 (Male = 60, Female = 64). ST = Small talk

### Moderation effect of gender on the relationship between DLPFC-TPJ synchronization and closeness-building metrics

**Fig. 4:**
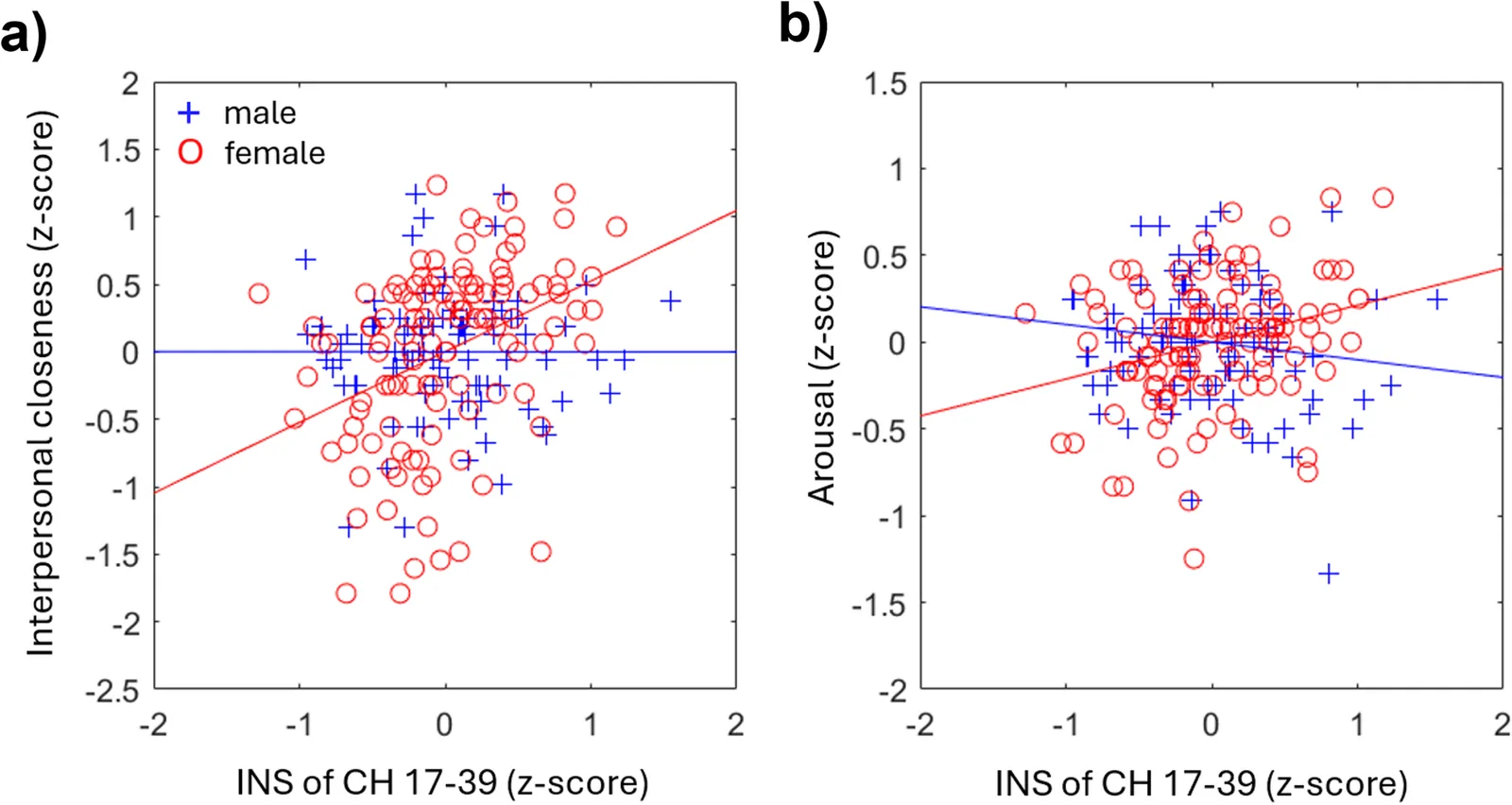
The moderation effect of gender group on the relationship between INS and behavioral measurements. Scatter plots depicting the association between INS (z-score) and **a** Interpersonal Closeness and **b** Emotional Arousal. Red circles and lines represent female dyads, while blue crosses and lines represent male dyads. The results indicate a significant positive correlation for female dyads, whereas no significant relationship was observed for male dyads

The linear mixture model analysis results revealed that the CH21-CH39 pair significantly predicted interpersonal closeness (*β* = 0.35, *p_*_*FDR*_ = .005, 95% CI = [0.15, 0.56]) and emotional arousal (*β* = 0.22, *p_*_*FDR*_ = .027, CI = [0.04 0.33]). Besides, we found a significant moderation effect of gender group on the relationship between ΔINS and interpersonal closeness (*β* = −0.39, *p_*_*FDR*_ = .025, 95% CI = [−0.68, −0.09]) and emotional arousal (*β* = −0.30, *p_*_*FDR*_ = .021, 95% CI = [−0.51, −0.10]). The moderation effect of gender was plotted in Fig. 4. For the female group, ΔINS at the CH21-CH39 pair significantly predicted the interpersonal closeness and emotional arousal (Interpersonal closeness*: β* = .32, *p* = .007, 95% CI = [0.09, 0.55]; Emotional arousal: *β* = 0.18, *p* = .011, 95% CI = [0.04, 0.33]). For the male group, ΔINS at the CH21-CH39 pair was not associated with the interpersonal closeness and emotional arousal (Interpersonal closeness: *β* = −0.03, *p* = .770, 95% CI = [−0.20, 0.15]; Emotional arousal: *β* = −.12, *p* = .133, 95% CI = [−0.27, 0.04]). The moderation effect analysis did not reveal significant results on the other two channel pairs (CH3-CH21 and CH17-CH39).

### The role of DLPFC-TPJ synchronization in mediating the association between self-disclosure and interpersonal closeness across genders

We further examined whether ΔINS could mediate the relationship between self-disclosure and other behavioral measurements, including interpersonal closeness, emotional arousal, and desire for further interaction. To reduce the possible effect of gender on behavioral measurements, we controlled for the effect of gender group and the interaction between gender group and ΔINS. The significant mediation model was only found in interpersonal closeness.

**Fig. 5:**
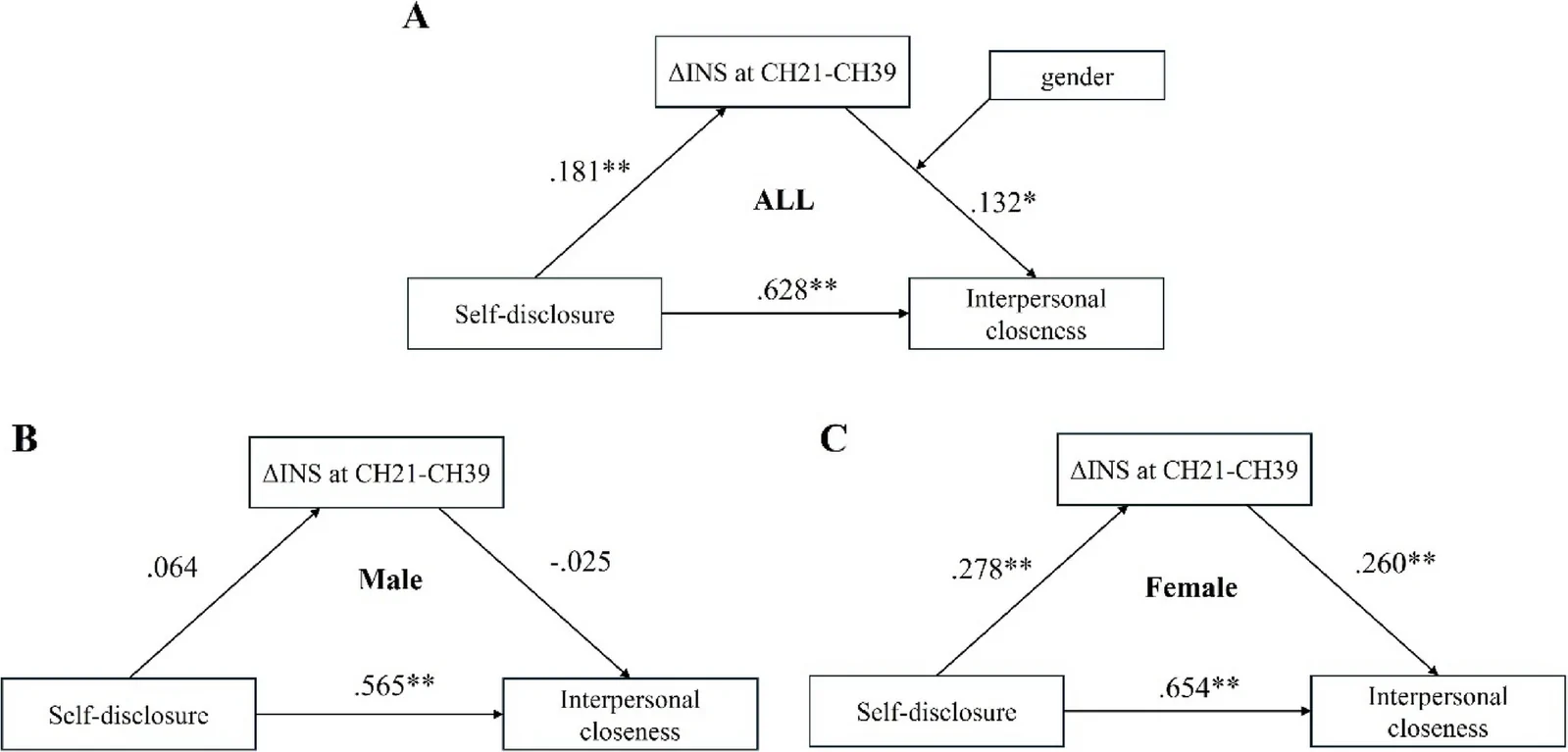
The moderated mediation model. **A** The ΔINS at CH21-CH39 mediated the effect of self-disclosure on interpersonal closeness, moderated by the gender group. The mediation model for (**B**) male and **C** female groups is also shown. Note: *: *p* <.05; **: *p* <.01; ***: *p* <.001

We assessed the mediation effect of INS on the relationship between self-disclosure and interpersonal closeness (Fig. 5). We found a significant *total effect* of self-disclosure on interpersonal closeness during the deep self-disclosure stage (Set 3) (*total effect* = 0.66, 95% CI = [0.51, 0.77]). ΔINS at CH21-CH39 channel pair was positively associated with self-disclosure (*β* = 0.17, *p* = .020, 95% CI = [0.04, 0.33]). Moreover, after controlling for the effect of ΔINS, self-disclosure was still a significant predictor for interpersonal closeness (*β* = 0.63, *p* < .001, 95% CI = [0.48, 0.74]), suggesting that the effect of ΔINS acted as a partial mediator of the relationship between self-disclosure on interpersonal closeness (*indirect effect* = .03, 95% CI = [0.00, 0.08]). Meanwhile, the gender group significantly moderated the relationship between ΔINS and interpersonal closeness. (gender × ΔINS: *β* = −0.26, *p* = .031, 95% CI = [−0.42, −0.02]).

We further assessed the mediation model on both gender groups separately. The results revealed that, only in the female group, the mediating effect of ΔINS was significant (Fig. 5).

## Discussion

While self-disclosure proved to be an effective method to enhance the relationship between strangers, the potential neural foundation was not fully understood. This study utilized fNIRS hyperscanning to delineate the INS mechanisms underlying self-disclosure and relationship building across genders. We found that the escalating depth of self-disclosure significantly increased interpersonal closeness, emotional arousal, and interpersonal attraction across the interaction stages. Uniquely for female dyads, this DLPFC-TPJ synchronization correlated with closeness and mediated the effect of self-disclosure on relationship formation, highlighting distinct neural interaction patterns between genders. Our work enriched the current understanding of how the relationship could be built for same-gender strangers, inspiring the development of treatment for the mental deficits that hinder social interaction ability.

### Escalated closeness and gender difference

Our results demonstrated that the increasing depth of self-disclosure manipulated through the structured tasks significantly enhanced perceived interpersonal closeness, aligning with previous research, which emphasizes the critical role of depth of self-disclosure in fostering interpersonal relationships [9, 55]. As the task stages progressed, all participants reported increased self-disclosure, interpersonal closeness, and emotional arousal. The gender effect analysis revealed a similar pattern of closeness improvement during the experiment. This proved the successful manipulation of dyads' interaction depth for both genders in our experiment.

However, the self-reported emotional arousal level of females and males significantly differed. Female-female dyads consistently showed higher levels throughout the four stages of the task. It may be influenced by gender-specific neurobiological and psychological mechanisms that enhance emotional engagement and responsiveness. Previous research has demonstrated that women generally show greater emotional responses in situations requiring emotional and empathetic involvement [56]. This enhanced emotionality can explain the sustained higher arousal levels in women observed during the experiment.

### DLPFC-TPJ cross-brain synchronization as a neural mechanism for relational closeness building

We found that closeness-building talk improved INS between DLPFC and TPJ. The DLPFC plays a vital role in social cognitive processes such as conflict resolution, belief formation, and evaluating norms and rules within social contexts [57–59]. The left DLPFC was also found to participate in the generation of language [60]. Meanwhile, the TPJ is engaged in social perception [61], notably in the theory of mind—the capability to attribute mental states to oneself and others [62], as well as empathic processing [63].

The coherence observed between the DLPFC and TPJ highlights a dynamic interplay between language generation and social perception during the closeness-building tasks. Because our conversational paradigm was characterized by naturalistic turn-taking, the participants constantly alternated between expressive and receptive roles. The speaker's DLPFC manages emotional self-disclosure and narrative production, while the listener's TPJ simultaneously processes this social information to facilitate empathy, intention reading, and mentalizing. This cross-brain inter-system loop matches recent advanced hyperscanning evidence showing cross-subject, cross-brain-region networks (asymmetric ATL–TPJ alignment) during affiliative bonding [50], reflecting how communication relies on synchronized interaction between one’s expressive production and the other’s receptive mentalizing networks. Previous hyperscanning studies have demonstrated that INS within the prefrontal cortex or the TPJ tracks interactive outcomes, such as cooperative performance [64]. Our findings extend this line of evidence by revealing that relationship formation relies not only on these localized intra-regional synchronies but also on a highly coordinated cross-brain-region network spanning the DLPFC and TPJ circuits.

### Gender-modulated divergences in the DLPFC-TPJ synchronization during conversation-driven friendship initiation

The moderation analysis revealed that the relationship between closeness, arousal, and INS was moderated by gender groups. Only the INS of female dyads, but not male dyads, significantly predicts the interpersonal closeness and arousal rating. This finding suggests that females were more likely to build close relationships with another female stranger through INS-enhancing conversation, compared to their male peers. Previous literature has documented that female-female dyads often elicit stronger or more functionally predictive inter-brain synchronization than male peers during social coordination. For instance, Baker et al. (2016) found that female pairs exhibited prominent task-related INS in the temporal cortex during cooperation [65], whereas male pairs showed synchronization primarily in prefrontal structures. Similarly, Zhang et al. (2017) revealed significant inter-brain coherence in the left temporoparietal junction (TPJ) exclusively within female-female dyads during social interactions [66]. This empirical divergence supports the notion that the conversation-driven synchronization observed across the DLPFC-TPJ pathway in our study acts as a critical, localized neural mechanism of relationship building tailored specifically to female interactive styles.

In our study, the gender differences in INS-induced closeness could also be explained by the higher emotional arousal of female-female dyads. The male-male dyads might suppress their emotional reaction to the intimate topic during the conversation, leading to less interpersonal closeness, although they might have INS as high as female-female dyads. This aligns with the previous finding that women may exhibit greater empathy and emotional sensitivity during the communication process [56].

Our following mediation analysis strengthened these findings regarding the gender differences—only in female-female dyads, we found the INS between DLPFC and TPJ significantly mediated the relationship between self-closure and interpersonal closeness. Specifically, the effect of INS on interpersonal closeness was not observed in male dyads, indicating that the INS during conversation might not be enough for males to build a relationship. This aligns with research characterizing female friendships by emotional sharing and male friendships by shared activities and competition [67, 68]. Evolutionarily, male cooperation often centered on agentic tasks (e.g., hunting) rather than emotional intimacy [32, 68], potentially explaining why INS during conversation did not predict closeness in male-male dyads in the current study. Although the observed magnitude of the mediation effect was small, these results provide primary evidence of the neural mechanism underlying the building process of close relationships.

### Limitations

Several limitations should be noted. First, the fixed, sequential task progression used to mimic real-life interaction inevitably confounded self-disclosure depth with time-dependent factors like habituation or fatigue. Although the distinct gender-specific dynamics observed under identical temporal conditions suggest our INS findings are driven by disclosure processes rather than mere time passage, future studies should employ counterbalanced designs to fully isolate intimacy depth from temporal factors. Second, without short-separation channels, potential systemic physiological artifacts cannot be completely excluded, particularly since our frequency of interest sits near the lower edge of typical human respiration. While the turn-taking nature of the paradigm and the high-threshold spatial PCA filter minimized the influence of global noise on the INS, future replication should integrate short channels to rigorously dissociate artifacts. Finally, our behavioral metrics relied entirely on subjective self-reports. Combining hyperscanning with concurrent autonomic indices or micro-level conversation feature analysis would enrich our understanding of the multi-modal dynamics driving relationship initiation.

### Conclusion

This study uncovered the INS mechanism underlying the closeness-building talk driven by self-disclosure. The INS between DLPFC and TPJ was found to be consistently improved during the self-disclosure talk. In the female-female interaction, the increase in INS also mediated the relationship between self-disclosure and interpersonal closeness. For males, the INS during conversation couldn’t predict their closeness level. From a perspective of evolution, this suggests that relationship formation between females may rely on emotional verbal expression more than their male peers. Understanding how gender influences these pathways could lead to effective strategies in various social settings in the future, from mental health therapies to workplace team dynamics.

## Supplementary Information

Supplementary Material 1.

## Data Availability

The datasets used and/or analysed during the current study are available from the corresponding author on reasonable request.
