# Supplementary material for: Gender-specific interpersonal neural synchronization during same-gender closeness-building interactions among strangers: an fNIRS hyperscanning study
Source: BMC Psychol. 2026 Aug 10;14:1365. doi: 10.1186/s40359-026-05129-6 (PMC13595777; doi:10.1186/s40359-026-05129-6)
Supplement: Supplementary file 1 [file 40359_2026_5129_MOESM1_ESM.docx]

**Gender-Specific Interpersonal Neural Synchronization During Same-gender Closeness-Building Interactions Among Strangers: An fNIRS Hyperscanning Study**

Yuhang Li ^1,2#^, Shibo Zhou ^2#^, Liutong Ou ^2#^, Yilin Hu ^2^, Xiuyun Lin ^3^, Qi Zhao ^1,2^, Rihui Li ^2,4^*, Peilian Chi ^1,2^*

1. Department of Psychology, Faculty of Social Sciences, University of Macau, Macau SAR, China
2. Centre for Cognitive and Brain Sciences, Institute of Collaborative Innovation, University of Macau, Macau SAR, China
3. Institute of Developmental Psychology, Faculty of Psychology, Beijing Normal University, Beijing, China
4. Department of Electrical and Computer Engineering, Faculty of Science and Technology, University of Macau, Macau SAR, China

*Correspondence concerning this article should be addressed to Rihui Li, and Peilian Chi at emails: [rihuili@um.edu.mo](mailto:rihuili@um.edu.mo) and [peilianchi@um.edu.mo](mailto:peilianchi@um.edu.mo).

#The authors contribute equally to the manuscript.

**S1. Exploratory Regression Analyses Predicting the Self-report Interaction States**

To explore the predictive boundaries of the identified Interpersonal Neural Synchronization (INS) patterns, we conducted a series of exploratory analyses with a linear mixed model. Specifically, we evaluated whether the task-evoked INS changes (Δ INS) during the deep self-disclosure stage (Set 3) could reliably predict participants’ interaction states, including “desire for further interaction”, “interpersonal closeness”, “self-disclosure”, “enjoyment during the interaction”, and “emotional arousal”.

Linear mixed models were constructed for the pooled sample, as well as separately for female-female and male-male dyads. The localized Δ INS values for the three key channel pairs of interest (CH3–CH21, CH17–CH39, and CH21–CH39) were entered as the predictor variables in separate blocks, with one of the interaction states serving as the dependent variable in each model. The dyad's ID was input as a random factor. As summarized in **Table S1**, besides the results reported in Sections 3.3 and 3.4 of the main text, the regression analyses did not yield any statistically significant predictive or correlational pathways within the overall sample or within either gender group (*p* > .05 for all models).

| Table S1: Linear Regression Results Using Task-Evoked INS (Set 3) to Predict Self-report Interaction States | | | | | | | | | | | |
| --- | --- | --- | --- | --- | --- | --- | --- | --- | --- | --- | --- |
| Group | Predictor (ΔINS) | Desire for further interaction desire | | Interpersonal closeness | | Self-disclosure | | Enjoyment during the interaction | | Emotional arousal | |
|  |  | *β* | *p*-value | *β* | *p*-value | *β* | *p*-value | *β* | *p*-value | *β* | *p*-value |
| Overall Sample | CH3 – CH21 | 0.08 | .538 | 0.13 | .177 | 0.15 | 0.134 | 0.02 | .850 | 0.10 | .162 |
|  | CH17 – CH39 | -0.03 | .800 | 0.10 | .336 | 0.08 | 0.464 | -0.05 | .535 | -0.02 | .764 |
|  | CH21 – CH39 | 0.14 | .283 | 0.35 | .001 | 0.23 | 0.037 | 0.16 | .081 | 0.22 | .011 |
|  |  |  |  |  |  |  |  |  |  |  |  |
| Female Dyads | CH3 – CH21 | 0.13 | .485 | 0.13 | .212 | 0.15 | 0.119 | 0.02 | .842 | 0.26 | .141 |
|  | CH17 – CH39 | 0.05 | .780 | 0.10 | .359 | 0.08 | 0.446 | -0.04 | .666 | 0.20 | .829 |
|  | CH21 – CH39 | 0.20 | .263 | 0.32 | .007 | 0.23 | 0.036 | 0.12 | .194 | 0.13 | .011 |
|  |  |  |  |  |  |  |  |  |  |  |  |
| Male Dyads | CH3 – CH21 | 0.02 | .914 | 0.01 | .943 | -0.17 | 0.194 | 0.00 | .970 | 0.09 | .264 |
|  | CH17 – CH39 | -0.12 | .528 | 0.10 | .222 | 0.05 | 0.645 | 0.00 | .978 | 0.08 | .204 |
|  | CH21 – CH39 | 0.05 | .776 | -0.03 | .770 | 0.07 | 0.536 | 0.04 | .652 | -0.11 | .134 |

**S2. The 46 Functional Inter-Brain Channel Pairs of Interest**

| Table S2 The 46 Functional Inter-Brain Channel Pairs of Interest | | | | | |
| --- | --- | --- | --- | --- | --- |
| No. | Channel Pair | No. | Channel Pair | No. | Channel Pair |
| 1 | CH1 - CH2 | 17 | CH6 - CH33 | 33 | CH16 - CH40 |
| 2 | CH1 - CH3 | 18 | CH6 - CH39 | 34 | CH17 - CH39 |
| 3 | CH1 - CH7 | 19 | CH8 - CH13 | 35 | CH17 - CH40 |
| 4 | CH1 - CH9 | 20 | CH8 - CH15 | 36 | CH21 - CH22 |
| 5 | CH1 - CH21 | 21 | CH8 - CH40 | 37 | CH21 - CH25 |
| 6 | CH1 - CH33 | 22 | CH10 - CH11 | 38 | CH21 - CH27 |
| 7 | CH3 - CH6 | 23 | CH10 - CH33 | 39 | CH21 - CH39 |
| 8 | CH3 - CH7 | 24 | CH10 - CH39 | 40 | CH21 - CH40 |
| 9 | CH3 - CH9 | 25 | CH12 - CH14 | 41 | CH22 - CH24 |
| 10 | CH3 - CH13 | 26 | CH12 - CH36 | 42 | CH22 - CH28 |
| 11 | CH4 - CH11 | 27 | CH13 - CH36 | 43 | CH25 - CH33 |
| 12 | CH4 - CH20 | 28 | CH14 - CH37 | 44 | CH25 - CH36 |
| 13 | CH5 - CH11 | 29 | CH16 - CH18 | 45 | CH25 - CH39 |
| 14 | CH5 - CH15 | 30 | CH16 - CH21 | 46 | CH25 - CH40 |
| 15 | CH6 - CH7 | 31 | CH16 - CH34 |  |  |
| 16 | CH6 - CH23 | 32 | CH16 - CH39 |  |  |
